# Genomic Prediction from Multi-Environment Trials of Wheat Breeding

**DOI:** 10.3390/genes15040417

**Published:** 2024-03-27

**Authors:** Guillermo García-Barrios, Leonardo Crespo-Herrera, Serafín Cruz-Izquierdo, Paolo Vitale, José Sergio Sandoval-Islas, Guillermo Sebastián Gerard, Víctor Heber Aguilar-Rincón, Tarsicio Corona-Torres, José Crossa, Rosa Angela Pacheco-Gil

**Affiliations:** 1 Postgrado en Recursos Genéticos y Productividad-Genética, Colegio de Postgraduados, Texcoco 56264, Estado de México, Mexico; garcia.guillermo@colpos.mx (G.G.-B.); sercruz@colpos.mx (S.C.-I.); aheber@colpos.mx (V.H.A.-R.); tcoro-nat@colpos.mx (T.C.-T.); 2 International Maize and Wheat Improvement Center (CIMMYT), Km 35 Carretera México-Veracruz, Texcoco 56237, Estado de México, Mexico; l.crespo@cgiar.org (L.C.-H.); p.vitale@cgiar.org (P.V.); guillermosgerard@gmail.com (G.S.G.); 3 Postgrado en Fitosanidad, Colegio de Postgraduados, Texcoco 56264, Estado de México, Mexico; sandoval@colpos.mx; 4 Posgrado en Socioeconomía Estadística e Informática, Colegio de Postgraduados, Texcoco 56264, Estado de México, Mexico

**Keywords:** bread wheat, genomic selection, factor analytic, epistatic variance

## Abstract

Genomic prediction relates a set of markers to variability in observed phenotypes of cultivars and allows for the prediction of phenotypes or breeding values of genotypes on unobserved individuals. Most genomic prediction approaches predict breeding values based solely on additive effects. However, the economic value of wheat lines is not only influenced by their additive component but also encompasses a non-additive part (e.g., additive × additive epistasis interaction). In this study, genomic prediction models were implemented in three target populations of environments (TPE) in South Asia. Four models that incorporate genotype × environment interaction (G × E) and genotype × genotype (GG) were tested: Factor Analytic (FA), FA with genomic relationship matrix (FA + G), FA with epistatic relationship matrix (FA + GG), and FA with both genomic and epistatic relationship matrices (FA + G + GG). Results show that the FA + G and FA + G + GG models displayed the best and a similar performance across all tests, leading us to infer that the FA + G model effectively captures certain epistatic effects. The wheat lines tested in sites in different TPE were predicted with different precisions depending on the cross-validation employed. In general, the best prediction accuracy was obtained when some lines were observed in some sites of particular TPEs and the worse genomic prediction was observed when wheat lines were never observed in any site of one TPE.

## 1. Introduction

Genomic selection is an alternative in breeding programs to address complex traits that are regulated by many genes that display minor effects [1]. Breeding value predictions are based on a covariance matrix that describes the additive relationship between the individuals considered [2]. However, an individual’s total genetic value is determined through a combination of additive and non-additive effects (dominance and epistasis) [3,4].

In quantitative genetics, epistasis refers to any interaction between genotypes in two or more loci [5]. If genotypes for the different loci display epistatic interactions, these result in a variance component, which is the variance of the epistatic interactions [6].

Epistatic variance is subdivided into components. First, a subdivision is carried out according to the number of loci involved: the two-factor interaction comes from the interaction of both loci, the three-factor interaction, from three loci, etc. The interactions involving a greater number of loci contribute so little to the variance that they can be ignored [6].

The subsequent subdivision of epistatic variance is based on whether the interaction involves breeding values or dominance deviations; thus, three types of two-factor interactions emerge. The interaction between the two breeding values results in additive x additive variance (V_AA_), the interaction between the breeding values of a locus and the dominance deviation of the other results in additive x dominance variance (V_AD_), and the interaction between the two dominance deviations results in the dominance x dominance variance (V_DD_). Thus, the epistatic variance splits into components in the following way: $V_{I}=V_{AA}+V_{AD}+V_{DD}+etc$., where the additional terms are similar components that rise from the epistatic interactions between over two loci [6].

Modeling additive x additive epistatic effects in genomic prediction can be restrictive because of the high computational burden caused by the large number of interactions among markers when all the interactions are considered [7,8]. An alternative to modeling epistasis and reducing the computational burden is to use models that include genomic relationship matrices as covariance structures for individuals [7].

Commonly, interactions between pairs of loci are modeled through regression coefficients of the Hadamard products of the genomic relationship matrix with itself. This approach has been implemented in several genomic prediction studies, covering wheat [7,9], sugar beet, rye [10], potato [11], *Pinus taeda* [12] and Japanese larch [13].

Initially, epistatic effects can be exploited in the theory of quantitative genetic selection [14]. However, estimating epistatic effects and their inclusion in breeding values have produced variable results in prediction accuracy.

In barley, Abed et al. [15] reported that modeling epistasis resulted in slight increases in the prediction accuracy of days to heading, days to maturity and plant height, but no advantages were evident for fusarium resistance traits, weight and grain yield.

Lorenzana and Bernardo [16] pointed out that including epistatic effects in prediction models resulted in a lower prediction accuracy compared to the models that only considered additive effects. This was observed in multiple *Arabidopsis*, maize and barley traits.

In research conducted by Wang et al. [17] in winter wheat, models incorporating marker and epistasis information were found to outperform models using marker information alone.

An aspect that has been scarcely explored is the epistatic interaction across environments. Multi-environment trials have been widely used in genetic plant breeding to evaluate genotype by environment interactions (G × E) and play an important role when selecting stable and high-yielding lines in different environments or lines adapted to local environmental constraints [18].

The aim of modeling epistasis and including multiple environments in genomic prediction is to achieve a greater prediction accuracy in comparison with the conventional models, which only consider additive effects. Hence, the intention of partially replacing costly experiments and/or save time, increasing the selection gain [9,19].

Multi-environment trials can be conducted across various sites and years, constituting a target population of environments (TPE). During evaluations, genotypes are grown under environmental conditions that differ among farms and years. These trials provide information to predict how a genotype will perform across the TPE [20,21].

Factor Analytic (FA) is a statistical method to model genotype × environment (G × E) interaction, adjusted within the framework of mixed models. Its implementation requires calculating the G × E covariance matrix (which represents the relationships between genotypes and environments). The FA model efficiently captures common patterns of G × E interaction [22,23,24,25].

The aims of this investigation were: (1) to predict the grain yield of bread wheat in three TPEs in South Asia and (2) to evaluate the predictive ability of four genomic prediction models: Factor Analytic (FA), Factor Analytic with a genomic relationship matrix (FA + G), Factor Analytic with an epistatic relationship matrix (FA + GG) and Factor Analytic with a genomic and epistatic relationship matrix (FA + G + GG).

## 2. Materials and Methods

### 2.1. Phenotypic Data

A total of 485 bread wheat genotypes, developed by the International Maize and Wheat Improvement Center (CIMMYT) were used, and distributed in 162 wheat cultivars, which were included in the target population of environment 1 (TPE1). 161 wheat cultivars were in tested TPE2 and 162 wheat lines were included in sites of TPE3. The TPEs in question are found in South Asia in India, Nepal and Bangladesh.

TPE1 contains six locations in India and one in Nepal. The sites in which the experiments were conducted were the Borlaug Institute for South Asia (Ludhiana, Punjab, India), Punjab Agricultural University (Ludhiana, Punjab, India), Indian Institute of Wheat and Barley Research (Karnal, Haryana, India), Indian Agricultural Research Institute (New Delhi, India), Chaudhary Charan Singh Haryana Agricultural University (Hisar, Haryana, India) and Khumaltar, Nepal.

TPE2 had three locations in India, one in Nepal and one in Bangladesh. The sites in which the experiments were conducted were Bihar Agriculture University (Bhagalpur, Bihar, India), (Kalyani, Bengala, India), (Siddharthanagar, Lumbini, Nepal), the Borlaug Institute for South Asia (Pusa, Bihar, India) and (Jamalpur, Bangladesh).

TPE3 was represented in three sites in India: The Agharkar Research Institute (Pune, Maharashtra, India), the Borlaug Institute for South Asia (Jabalpur, Madhya Pradesh, India) and the Indian Agricultural Research Institute (Indore, Madhya Pradesh, India).

Yield evaluations were conducted between 2021 and 2023, using an alpha lattice experimental design with two repetitions. For more details of the grain yield evaluation methodology, see Pietragalla and Pask [26]. Usually sites and wheat breeding lines for each TPE change from year to year.

The wheat lines were genotyped using the genotyping-by-sequencing method [27], and libraries were sequenced using the Illumina NovaSeq 6000 (Illumina, CA, USA). SNP calling was conducted using the system patented by DArT P/L (Canberra, Australia).

SNP marker matrix was transformed to the numeric format 1, 0, −1, where 1 indicates the individual is homozygous for the most frequent allele, 0 denotes heterozygosity and −1 indicates homozygosity for the least frequent allele. The matrix marker was further edited and updated applying the standard norms of quality control by removing markers with over 40% missing data and minor allele frequencies lower than 5%. The missing data were imputed using the expectation–maximization algorithm. From this marker data the genomic similarity matrix (G) matrix was developed and used for the genomic prediction.

### 2.2. Statistical Models

Implementing the mixed model was carried out following Crossa et al. [28]:

$$Y=Xb+Z_{r}r+Z_{g}g+Z_{ge}ge+e$$

where Y is the vector of phenotypic observations, X, $Z_{r}$, $Z_{g}$ and $Z_{ge}$ are the design matrices for the fixed effects of sites, random effects of replications within sites, genotypes and G × E interaction, respectively. Vector b denotes the fixed effects of sites and vectors r, g, ge and e contain the random effects of replications within sites, genotypes, genotype x environment interaction, and residuals, respectively. 

The assumption is made that r, g, ge and e are random and normally distributed, with zero-mean vectors and variance-covariance matrices *R*, *G*, *GE* and *E*, respectively, such that:

$$\left[\begin{array}{c} r\\ g\\ ge\\ e \end{array}\right]\sim N\left(\left[\begin{array}{c} 0\\ 0\\ 0\\ 0 \end{array}\right],\left[\begin{array}{cccc} R & 0 & 0 & 0\\ 0 & G & 0 & 0\\ 0 & 0 & GE & 0\\ 0 & 0 & 0 & E \end{array}\right]\right)$$

The variance in *Y* is $V\left(Y\right)=Z_{r}\;RZ_{r}^{\prime }+Z_{g}GZ_{g}^{\prime }+Z_{ge}GEZ_{ge}^{\prime }+E$. Under the assumption, the R and E variance-covariance matrices have a simple variance component structure.

$$R=\Sigma_{r}⊗I_{r}=\left[\begin{array}{cccc} \sigma_{r_{1}}^{2} & 0 & \ldots & 0\\ 0 & \sigma_{r_{2}}^{2} & \ldots & 0\\ \vdots & \vdots & \ddots & \vdots \\ 0 & 0 & \ldots & \sigma_{r_{s}}^{2} \end{array}\right]⊗I_{r\prime }$$

$$E=\Sigma_{e}⊗I_{rg}=\left[\begin{array}{cccc} \sigma_{e_{1}}^{2} & 0 & \ldots & 0\\ 0 & \sigma_{e_{2}}^{2} & \cdots & 0\\ \vdots & \vdots & \ddots & \vdots \\ 0 & 0 & \ldots & \sigma_{e_{s}}^{2} \end{array}\right]⊗I_{rg}$$

where r is the number of repetitions, g is the number of genotypes, $I_{r\prime }$ and $I_{rg}$ are the identity matrices of orders r and rg, respectively. $\Sigma_{r}$ and $\Sigma_{e}$ are the repetition and residual variance matrices, respectively. ⨂ is the Kronecker product of both matrices. The residuals are assumed to have a normal multivariate distribution normal with a mean of zero and an *E* covariance matrix.

In order to model the terms *G* and *GE* in terms of some hypothetical factors, the factor analytic structure was used, which consisted in: suppose that the effect of the unobservable factor of the *i*-th genotype in the *j*-th environment can be expressed as $\sum_{k=1}^{t}\delta_{ik}x_{jk}+d_{ij}$, where $\delta_{ik}$ is the *k*-th random regression coefficient of the *i*-th genotype; $x_{jk}$ is the *k*-th latent variable related to the *j*-th site and $d_{ij}$ is the residual of the end of the interaction. In matrix terms, the vector of genotypic effects is represented by g=∆x+d, so the variance-covariance matrix of *g* is $V\left(g\right)=∆V\left(x\right){∆}^{\prime }+D$ and given that $V\left(g\right)=I$, then $V\left(g\right)=∆{∆}^{\prime }+D$. With this, one can define that the structure of the analysis of factors with *q* ≤ *e* factors (or components) (FA(*q*)) is in the form $∆{∆}^{\prime }+D$, where ∆ is an *e* × *q* matrix of the δ′s and *D* is the positive diagonal matrix *e* × *e* [29].

The implemented models were named FA, FA + G, FA + GG and FA + G + GG. FA model was the simplest and was built with grain yield data at different sites and considering the G × E interaction. The additive model FA + G included the genomic relationship matrix derived from markers in the form:

$$G_{A}=\frac{\left(X-\mu_{E}\right){\left(X-\mu_{E}\right)}^{T}}{2\sum_{j=1}^{p}p_{j}\left(1-p_{j}\right)}$$

where X is the SNP marker matrix of dimensions *j* × *p*. $p_{j}$ is the minor allele frequency of SNP j=1,…,p and $\mu_{E}$ is the expected value of matrix X under the Hardy–Weinberg equilibrium.

FA + GG model considers the additive x additive epistatic effects. The first order epistatic relationship matrix was computed using the Hadamard product (cell-by-cell multiplication) $G_{A}$#$G_{A}$.

The most comprehensive model was FA + G + GG, which includes the genomic relationship matrices and the additive × additive epistatic relationship matrices. These two matrices were constructed using the sommer package [30] in R [31]. The models were adjusted using the software ASReml-R 4.1 [32].

### 2.3. Cross-Validation Schemes

Three cross-validation schemes (CV) were implemented. CV1 comprised predicting the breeding value of genotypes that have not been evaluated; 80% of the genotypes were used for training and 20% for testing. In CV2, the breeding value of genotypes that have been evaluated in some sites but not in others was predicted. The training and testing sets consisted of disjoint sets of genotypes. CV3 comprised predicting the breeding value only at one site, with 10% of genotypes for training and the remaining 90% for testing; at other sites, all genotypes were included in the training set. Table 1 contains an example of the number of genotypes used for training and testing under the validation schemes in TPE1 sites.

Each model’s accuracy was determined by the average of the Pearson correlation between the predicted and observed breeding values of the test set in the 5-fold. The results were plotted using the R package ggpubr [33].

## 3. Results

### 3.1. Descriptive Yield Statistics in the TPEs

The average grain yield in TPE1 was 5.88 ± 0.4 t ha^−1^. The yield correlations between sites were variable, from 0.9 (between Karnal and New Delhi) down to an absence of correlation (0.12) between Ludhiana (PAU) and Ludhiana (BISA) (Table 2).

In TPE2, the average grain yield obtained was 4.47 ± 0.27 t ha^−1^. The yield correlations between sites were positive and moderate, fluctuating between 0.5 and 0.69 (Table 3).

In TPE3, the average grain yield was 5.74 ± 0.29 t ha^−1^. The locations of Indore and Jabalpur had a correlation of 0.24, whereas there was an absence of correlation between the locations of Pune and Indore, Indore and Jabalpur (Table 4).

### 3.2. Prediction of the Breeding Value of Non-Evaluated Genotypes (Cross-Validation CV1)

**Figure 1:**
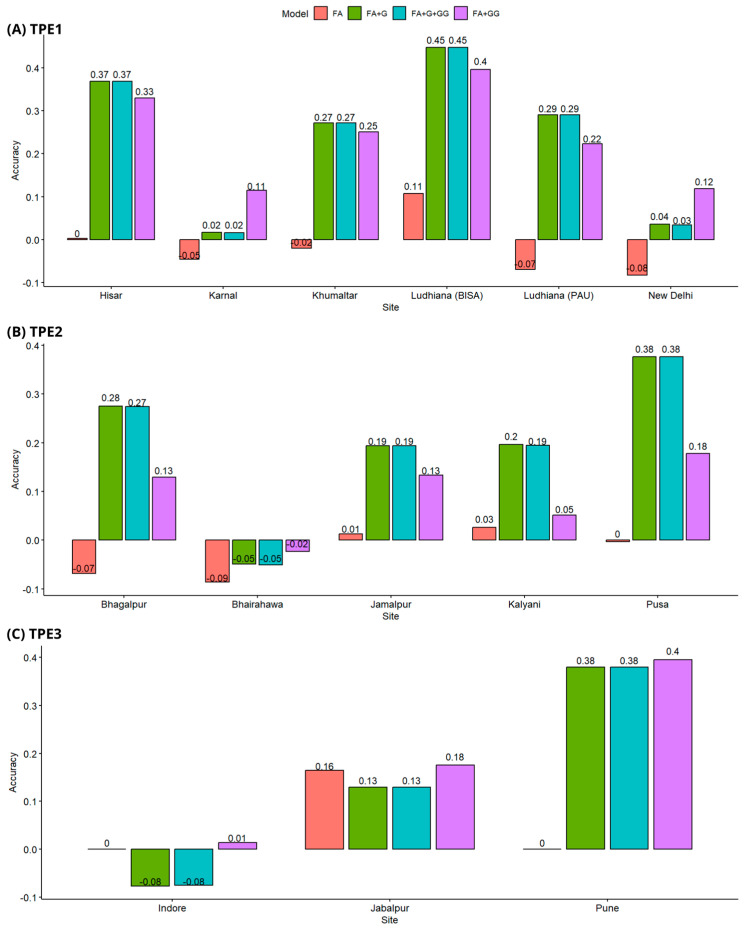
Prediction accuracy scheme CV1, (**A**) Predictive accuracy in TPE1, (**B**) Predictive accuracy in TPE2, (**C**) Predictive accuracy in TPE3. Models Tested: FA (Factor Analytic), FA + G (FA with genomic relationship matrix), FA + GG (FA with epistatic relationship matrix), FA + G + GG (FA with genomic and epistatic relationship matrices).

In TPE1, the sites with the best prediction accuracy were Ludhiana (BISA) and Hisar. The models with the best performance for these sites were FA + G y FA + G + GG, with prediction accuracies ranging from 0.45 ± 0.06 to 0.37 ± 0.12. The sites with the lowest accuracies were New Delhi and Karnal. For these two sites, the epistatic model FA + GG had the best performance. The model with the worst average performance in TPE1 was FA (Figure 1A).

In TPE2 the models with the best performance were also FA + G y FA + G + GG, with accuracies that ranged from 0.19 ± 0.14 for Kalyani to 0.19 ± 0.17 for Jamalpur to 0.38 ± 0.13 for Pusa. The site of Bhairahawa had negative predictions for all models (Figure 1B).

In TPE3, the location of Pune had the best predictions, with epistatic model FA + GG having the best performance with an accuracy of 0.4 ± 0.06. For the location of Indore, no model achieved acceptable predictions. The model with the lowest performance in TPE3 was FA, which did not adjust due to having insufficient degrees of freedom (Figure 1C).

### 3.3. Prediction of Breeding Value in Genotypes Evaluated in Some Environments, but Not in All (Cross-Validation CV2)

**Figure 2:**
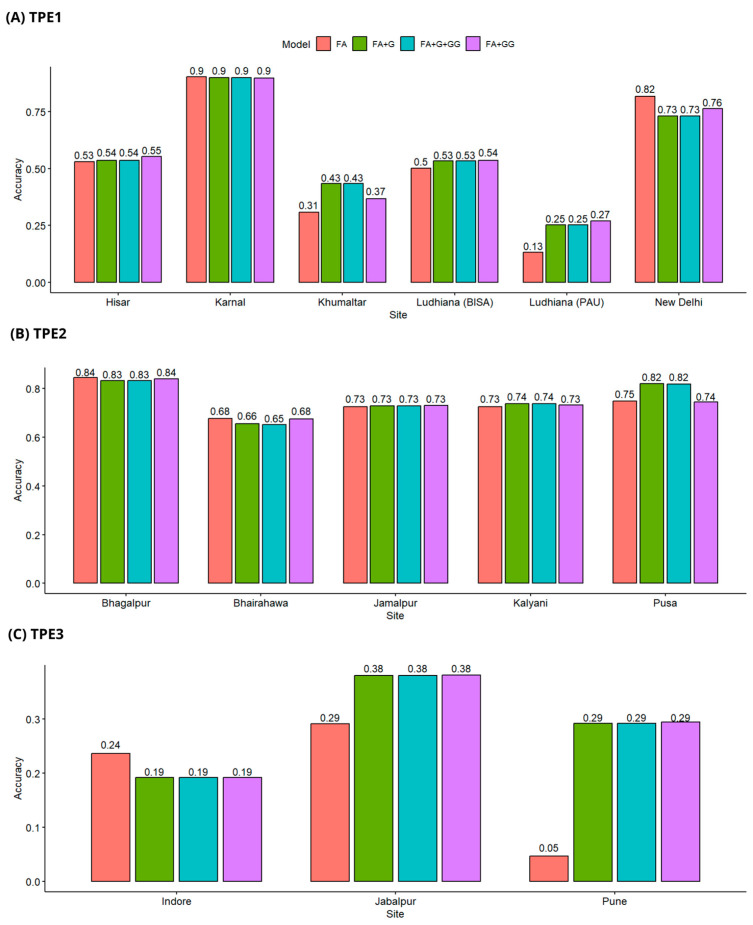
Prediction accuracy scheme CV2, (**A**) Predictive accuracy in TPE1, (**B**) Predictive accuracy in TPE2, (**C**) Predictive accuracy in TPE3. Models Tested: FA (Factor Analytic), FA + G (FA with genomic relationship matrix), FA + G + GG (FA with genomic and epistatic relationship matrices), FA + GG (FA with epistatic relationship matrix).

In predicting the breeding values of genotypes that have been evaluated in some environments, but not in all, the FA + G and FA + G + GG models had similar performances across all TPEs (Figure 2).

In TPE1, the site of Karnal had the best predictions, reaching the highest accuracy of 0.90 ± 0.06 with the four models tested. The site with the worst predictions was Ludhiana (PAU), with a maximum accuracy for this site being 0.27 ± 0.19 (Model FA + GG), and the lowest being 0.13 ± 0.14 (Model FA) (Figure 2A).

TPE2 had a better prediction accuracy than TPE1 and TPE3 under the CV2 validation scheme. The four models tested in TPE2 had a consistent performance across all sites, with the highest precision being 0.84 ± 0.06, corresponding to the FA and FA + GG models in the site of Bhagalpur, and the lowest precision 0.65 ± 0.11 for models FA + G and FA + G + GG in the site of Bhairahawa (Figure 2B).

In TPE3, models FA + G, FA + GG and FA + G + GG had a consistent performance, obtaining the same prediction accuracy within sites. FA had a variable performance, being the worst model for the site of Pune (0.05 ± 0.18 accuracy), yet this model achieved the highest precision for the site of Indore (0.24 ± 0.24) (Figure 2C).

### 3.4. Predicting the Breeding Value Using 90% of Genotypes for Training (Cross-Validation CV3)

**Figure 3:**
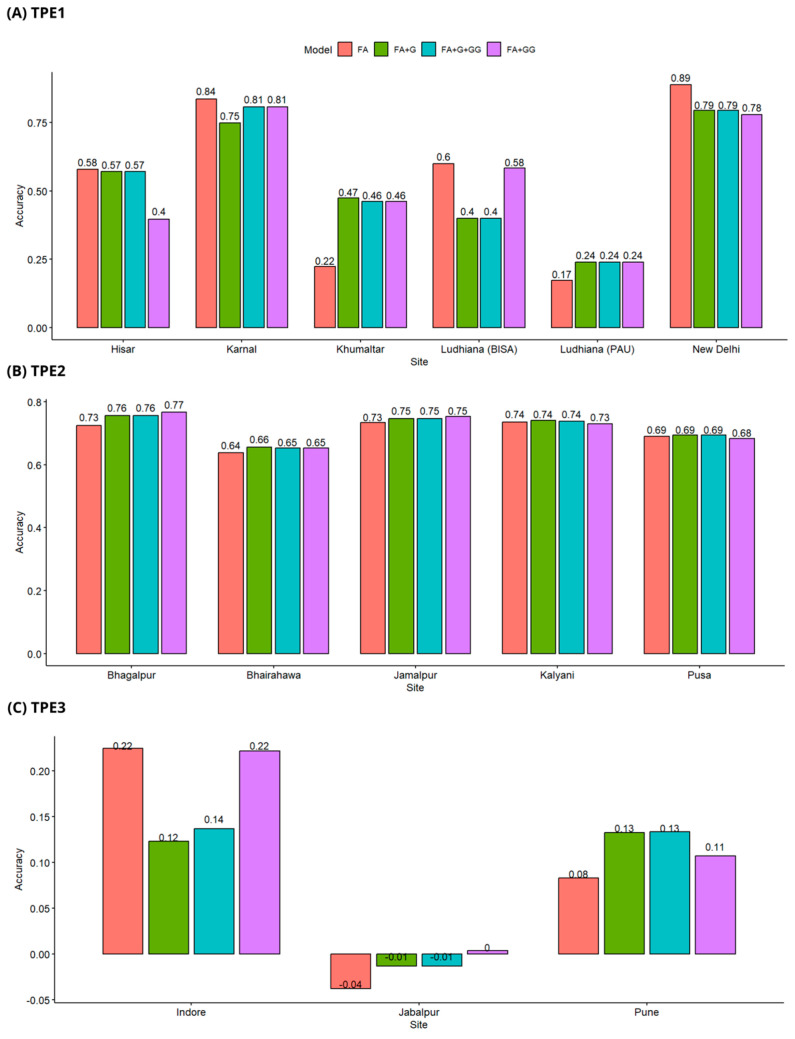
Prediction accuracy scheme CV3, (**A**) Predictive accuracy in TPE1, (**B**) Predictive accuracy in TPE2, (**C**) Predictive accuracy in TPE3. Models Tested: FA (Factor Analytic), FA + G (FA with genomic relationship matrix), FA + G + GG (FA with genomic and epistatic relationship matrices), FA + GG (FA with epistatic relationship matrix).

In the validation scheme CV3, models FA + G and FA + G + GG had a similar performance in the three TPEs, with similar prediction precisions. FA had an acceptable performance in TPE1 and TPE2, which contain six and five sites, respectively, although this same model had a bad performance in TPE3, which is composed of three sites (Figure 3).

In TPE1, the sites that had the best predictions were New Delhi and Karnal, with the highest precisions being 0.89 and 0.84, respectively, with the model FA. The site of Ludhiana (PAU) had the worst prediction accuracies, with values of 0.17 for model FA and 0.24 for the rest of the models (Figure 3A).

In TPE2, the four models tested had an acceptable performance and there was minor variation between them. The highest prediction accuracy was 0.77, which corresponds to the site of Bhagalpur and the epistatic model FA + GG. The lowest accuracy was 0.64, which corresponds to the location of Bhairahawa and the model FA (Figure 3B).

Out of the three environments evaluated, TPE3 displayed the worst accuracies. The site of Indore reached the highest accuracies (0.22) with models FA and FA + GG (Figure 3C).

## 4. Discussion

### 4.1. Including Epistasis in the Genomic Prediction Models of Bread Wheat Does Not Increase Prediction Accuracy

In this study, we evaluated four prediction models that considered the additive and epistatic effects, as well as their combination. The model that included the additive and epistatic effects (FA + G + GG) was expected to have a higher prediction accuracy than the rest of the models, although FA + G + GG had a similar performance to the additive model FA + G in almost all tests.

Mackay [5] establishes a difference in the additive genetic variance, considering it “real” when most of the loci that affect the trait have additive genetic action, or “apparent” when the main non-zero effects arise from the action of the epistatic gene in many loci. Under this distinction, we can suggest that our additive model could capture some of the epistatic effects; therefore, the additive (FA + G) and additive + epistatic (FA + G + GG) models displayed similar performances.

The average effects of individual loci may capture a portion of epistasis, implying that interaction effects between loci become “apparent” additive effects; this is more likely to happen when allele frequencies approach 0 or 1 due to genetic drift or selection [34,35,36].

In a study conducted on *P. taeda* by Muñoz et al. [12] modest variations in the Akaike information criterion were obtained when comparing additive models and models that simultaneously considered additive and non-additive effects. The authors suggest additive models capture part of the genetic variation, due to the dominance and epistasis and that the additive model should not suffer much if these components are omitted.

Dong et al. [13] implemented genomic prediction models related to growth, physical and chemical properties in wood in *Larix kaempferi*. Their results showed that the traditional GLUP model performed similarly to GLUP with non-additive effects. These results coincide with those obtained in this study.

A study by Wang et al. [17] presented opposite results to ours. They reported that the consideration of epistatic effects on the genomic prediction models resulted in a higher prediction accuracy. However, this study was conducted considering only the effects of major markers and ignores that epistasis is not limited to interactions between loci associated with the trait, but interactions also occur with loci that have no significant effects on the traits when evaluated individually.

Wientjes et al. [37] conducted a simulation study on a livestock population where they evaluated genomic prediction models. In the first generation of selection, the accuracy of the additive and additive + dominance models was ~0.83, while it was ~0.72 for the model incorporating additive, dominance, and epistatic effects. These results are consistent with those observed in our study, wherein the inclusion of additional factors in the model did not lead to improved predictive accuracy.

### 4.2. Cross-Validation Schemes

Having information about performing a line across multiple sites makes it easier to predict its performance at a new site, while predicting the performance of a newly developed line (with no history) is more challenging and requires using the performance of similar genotypes for predictions. This is supported by our results, where the CV2 validation scheme had an advantage over CV1.

In TPE2, under the CV2 and CV3 scheme, prediction accuracies ranged from 0.64 to 0.84. As a general rule, a correlation of 0.5 is acceptable, given that prediction accuracy is the correlation between the phenotypic value and the predicted breeding value, it is common for accuracy to be downward biased because the phenotype is an imperfect estimator of the breeding value [38].

The good accuracy accomplished in TPE2 with the CV2 and CV3 scheme is possibly an indicator that genomic selection can be implemented in this TPE, which would allow for the reduction in costly experiments and increase selection gains. Further studies in TPE2 may include evaluations over seasons or years.

The advantage of the CV3 validation scheme over CV1 may be attributed to the larger size of the training set in CV3, thus capable of capturing more variance than a small training set and therefore generating high prediction accuracies.

### 4.3. Advantages of the Factor Analytic Model for Studying Genotype × Environment Interaction in the Context of Genomic Prediction

The FA offers flexibility in capturing the underlying structure of G × E interaction. It allows for the estimation of latent factors that represent the main patterns of G × E interaction, which can be beneficial for understanding the genetic basis of complex traits and designing breeding strategies. Also, FA identifies latent factors associated with G × E interaction. This can help breeders understand the genetic architecture of traits and make informed decisions regarding genotype selection and environmental management. However, FA may require more computational resources compared to simpler models. Efficient algorithms and parallel computing techniques can help improve the computational efficiency of FA.

## 5. Conclusions

It was confirmed that additive models capture part of the epistatic variance, showing the suitability of the FA + G model to include in genomic selection programs. Our research also corroborates the efficiency of the Factor Analytic model to capture G *×* E interactions, particularly when integrating information from multiple sites. Furthermore, our results indicated that the FA model exhibited good performance when using data from six to five sites (TPE1 and TPE2, respectively); however, it performed poorly in TPE3, which contained only three sites.

## Figures and Tables

**Table 1:** Example of the number of genotypes used in the training and testing set for 1-fold. In CV1, the breeding value of 32 genotypes was predicted across six locations. CV2 used prior performance data from Ludhiana (PAU), New Delhi, and Khumaltar to predict the performance of 32 genotypes at Ludhiana (BISA), Karnal, and Hisar. In CV3, the breeding value of 142 genotypes was predicted in Ludhiana (BISA).

Cross-Validation Schemes	Set	Ludhiana (BISA)	Ludhiana (PAU)	Khumaltar	Karnal	New Delhi	Hisar
CV1	Training	130	130	130	130	130	130
	Testing	32	32	32	32	32	32
CV2	Training	130	162	162	130	162	130
	Testing	32	0	0	32	0	32
CV3	Training	20	162	162	162	162	162
	Testing	142	0	0	0	0	0

**Table 2:** Yield correlation between sites of TPE1.

	Ludhiana (BISA)	Ludhiana (PAU)	Khumaltar	Karnal	New Delhi	Hisar
Ludhiana (BISA)	1					
Ludhiana (PAU)	0.12	1				
Khumaltar	0.20	0.00	1			
Karnal	0.59	0.14	0.21	1		
New Delhi	0.55	0.18	0.23	0.90	1	
Hisar	0.35	0.18	0.48	0.42	0.48	1

**Table 3:** Yield correlation between sites of TPE2.

	Bhagalpur	Jamalpur	Pusa	Bhairahawa	Kalyani
Bhagalpur	1				
Jamalpur	0.69	1			
Pusa	0.63	0.64	1		
Bhairahawa	0.57	0.59	0.50	1	
Kalyani	0.69	0.64	0.60	0.62	1

**Table 4:** Yield correlation between sites of TPE3.

	Pune	Indore	Jabalpur
Pune	1		
Indore	0.09	1	
Jabalpur	−0.04	0.24	1

## Data Availability

The data utilized in this study, encompassing both phenotypic and genomic information, are available for download from the following link. https://github.com/guillermogarci4/MultiEnv-Wheat-Genomic-Prediction.git (accessed on 1 March 2024).
